# Modeling Behavior and Vaccine Hesitancy Using Twitter-Derived US Population Sentiment during the COVID-19 Pandemic to Predict Daily Vaccination Inoculations

**DOI:** 10.3390/vaccines11030709

**Published:** 2023-03-22

**Authors:** Talal Daghriri, Michael Proctor, Sarah Matthews, Abdullateef H. Bashiri

**Affiliations:** 1 Department of Industrial Engineering, Jazan University, Jazan 82822, Saudi Arabia; 2 Department of Industrial Engineering & Management Systems, University of Central Florida, Orlando, FL 32816, USA; 3 Interdisciplinary Modeling and Simulation Program, University of Central Florida, Orlando, FL 32816, USA; 4 Department of Mechanical Engineering, Jazan University, Jazan 82822, Saudi Arabia

**Keywords:** COVID-19 vaccination, vaccine hesitancy, Twitter & CDC datasets, sentiment analysis, daily vaccine inoculations

## Abstract

The sentiment analysis of social media for predicting behavior during a pandemic is seminal in nature. As an applied contribution, we present sentiment-based regression models for predicting the United States COVID-19 first dose, second dose, and booster daily inoculations from 1 June 2021 to 31 March 2022. The models merge independent variables representing fear of the virus and vaccine hesitancy. Large correlations exceeding 77% and 84% for the first-dose and booster-dose models inspire confidence in the merger of the independent variables. Death count as a traditional measure of fear is a lagging indicator of inoculations, while Twitter-positive and -negative tweets are strong predictors of inoculations. Thus, the use of sentiment analysis for predicting inoculations is strongly supported with administrative events being catalysts for tweets. Non-inclusion in the second-dose regression model of data occurring before the 1 June 2021 timeframe appear to limit the second-dose model results—only achieving a moderate correlation exceeding 53%. Limiting tweet collection to geolocated tweets does not encompass the entire US Twitter population. Nonetheless, results from Kaiser Family Foundation (KFF) surveys appear to generally support the regression factors common to the first-dose and booster-dose regression models and their results.

## 1. Introduction

From June 2011 to April 2019, access to commentary by individuals on social media enabled researchers to study the controversial influence on public sentiment and behavior generated through global social media channels with a particular focus on vaccine hesitancy [1]. Other researchers in Korea [2], Turkey [3], India [4], and the United States of America [5,6] expanded social media sentiment analysis; in particular, using Twitter tweets as a significant resource of data and analysis to rapidly track and quantified public opinions, beliefs, or behavior regarding critical events, pandemic-related events, personalities, or subjects including quickly and effectively measuring vaccine hesitancy.

In the early stages of the COVID-19 pandemic, online contributors put forth predictions through various social media channels with one of the more infamous declarations of an “eradication” phase ending the pandemic in June 2021 [7]. Like so many other commentaries through social media during the pandemic, the prediction was proven wrong despite its widespread distribution and possible influence on the public. Staying abreast of rapidly changing COVID-19 events during the first half of 2021 [8,9], inferred that similar levels of social media positive and negative sentiments toward vaccines indicated that proportionally equal segments of the population were either inclined or not inclined toward being vaccinated. Declining cases and deaths from 14 January 2021 until 23 June supported the “eradication” prediction, leading to a decrease in fear of the virus [10]. The US situation changed rapidly with the emergence of the Delta variant. The sudden increase in the number of cases, the severity of symptoms as evidenced by increasing deaths, and changing social sentiment appeared to change behavior toward vaccine acceptance and inoculation. Researchers similarly extended vaccine social media sentiment analysis [6,7,8].

The health belief model (HBM) asserts that people’s particular beliefs, such as their perceptions of the disease’s severity and susceptibility as well as the benefits and risk of vaccination, are related to their behavior in terms of their health [11,12,13]. The notion that acquiring (contracting) the disease will have severe consequences for both the patient and others is referred to as perceived severity (i.e., the absolute risk) or the level of “fear of the virus”. People who view themselves as being in danger or as having a high risk of contracting COVID-19 are more likely to express strong intentions to get the COVID-19 vaccine [14]. The level of fear of post-vaccination side effects is a significant factor in determining the level of vaccine hesitancy among significant segments of the public [13].

## 2. Statement of Contribution

**Figure 1:**
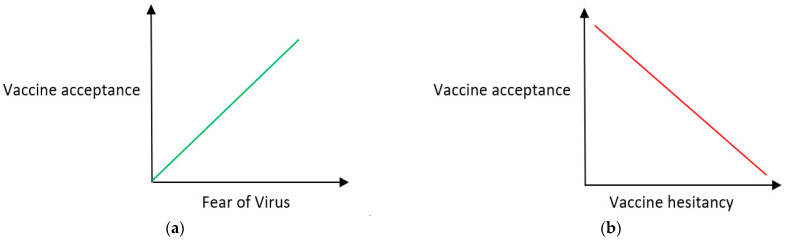
(**a**): Fear of Virus and Vaccinations Acceptance. (**b**): Vaccine Hesitancy and Vaccine Acceptance.

This research starts with two premises, the first being that the level of vaccine acceptance or inoculation is driven by the level of fear of the virus with higher fear-of-virus threats (e.g., illness, death), theoretically resulting in increasing vaccine acceptance or increasing inoculation (Figure 1a). The second premise is that the level of vaccine acceptance or inoculation is also driven by the level of fear of vaccine side effects resulting in vaccine hesitancy, with higher vaccine hesitancy theoretically resulting in a decrease in vaccine acceptance and a decrease in inoculations (Figure 1b) [15].

This research merges these two inconsistent drivers of inoculation behavior into data-driven models. The resulting models extend social media sentiment analysis by applying regression to predicting the United States daily vaccine inoculations during the research timeframe of 1 June 2021 to 31 March 2022. Spanning this timeframe, this research creates predictive regression models for each of the three vaccine inoculation types—first dose, second dose (fully vaccinated), and booster. The models encompass the three phases of the COVID-19 pandemic in the United States during this timeframe including a portion of the errant COVID-19 “eradication phase” as the Baseline phase, the Delta variant phase, and the Omicron variant phase. The regression research approach uniquely incorporates positive and negative sentiment analysis of virus fear and vaccine hesitancy from Twitter tweets supplemented by CDC data to predict future CDC first dose, second dose, and booster inoculations.

Further, the research indicates the degree to which different fears, factors, and levels impact the daily vaccine inoculation count by segmenting the pandemic timeframe into phases based on CDC VOC announcements. The research revealed that each phase impacted the vaccine inoculation models based on phase characteristics and events and the public response to those characteristics and events. Inconsistent correlations between traditional indicators of fear of the virus and traditional indicators of fear of the vaccine were also accompanied by rapid changes in the vaccine inoculation trend. Likewise, first-, second-, and booster-dose inoculations’ perceived value vs. risks during a phase impacted the vaccine inoculation models. In order to more accurately quantify and predict inoculation trends, the research extends Twitter sentiment analysis by classification and quantification of the nature and strength of the association between opinions on Twitter and daily vaccination and inoculation spikes. Overall, the regression models provide the means for predicting first-, second-, and booster-dose daily vaccination inoculation in the USA. Regression results are consistent with KFF vaccination opinion surveys and with the Technology Acceptance Curve [16]. Comments on limitations and future research goals are provided.

## 3. Materials and Methods

To predict vaccine inoculations in light of fear of the virus and vaccine hesitancy, this research methodology used three primary US population datasets. First, the Center of Disease Control (CDC) identifies virus and variant threats relevant to the US population through virus alerts and subsequent Variant of Concern (VOC) alerts [17]. The FDA in coordination with the CDC approves vaccines and associate vaccine inoculation guidelines for state and local health agencies. [18]. The CDC reports daily virus cases (a traditional measure of the level of threat of becoming sick from the virus), virus deaths (a traditional measure of the level of threat of dying from the virus), and inoculations (a traditional measure of dose acceptance) [19]. The CDC also identifies COVID-19 treatments and medications used to mitigate virus effects but does not report daily outcomes [20]. In terms of vaccine side effects, the CDC also reports “Selected Adverse Events Reported after COVID-19 Vaccinations” but the reports are not a daily occurrence, have significant latency between events and reports, and do not claim to represent a collection of all adverse events [21]. As a supplemental measure to account for CDC virus mitigation and vaccine side effect reporting limitations and as demonstrated previously by [6].

Techniques for accessing social media vary. Our method involved the use of a Twitter-provided database of millions of users’ data who previously released their data to Twitter. Twitter provided the data to the approved researchers in a format without personally identifiable information and did not require engagement with users for access, thus avoiding the federally regulated Institutional Review Board review. The sentiment analysis of Twitter users’ geolocated tweets, while limited [8,22], may be used to identify levels of fear of the virus and levels of vaccine hesitancy in a given population during a pandemic. To explain, geolocated tweets do not represent all Twitter populations as not all users provide their location information. Furthermore, given the linguistic diversity of the USA, another limitation of our study is that we only looked at English-language tweets [22,23]. Twitter’s API provides access to 1% of the public tweets by random sampling in near real-time. Despite the potential concern about biased or imbalanced data for collecting only 1% of all tweets, it has been demonstrated that sentiments found from API samples and the full tweet dataset shows the same sentiment levels with very small deviation (1.8%) [8,24]. 

**Figure 2:**
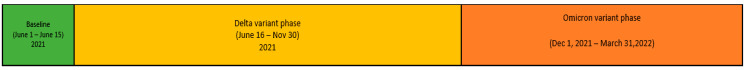
Pandemic Phases within the Research Timeframe.

Within the 1 June 2022 to 31 March 2022 timeframe, data collection focuses on three pandemic phases determined by the CDC virus and VOC alerts (“SARS-CoV-2 B.1.617.2 (Delta) variant COVID-19 outbreak...”, 2021; “Coronavirus disease 2019 (COVID-19)”, 2022) (Figure 2). The Baseline phase encompasses 1–15 June 2021 and represents the state of fear caused by the COVID-19 virus and as mitigated by the existing vaccines just prior to the CDC alerting the public on 15 June 2022 of the Delta VOC. The Delta variant phase follows the Delta VOC alert and spans the period from June 16th to CDC Omicron VOC issued on 30 November 2022. The Omicron variant phase follows the Omicron VOC alert on 1 December 2021 until the end of the research study on 31 March 2022.

**Figure 3:**
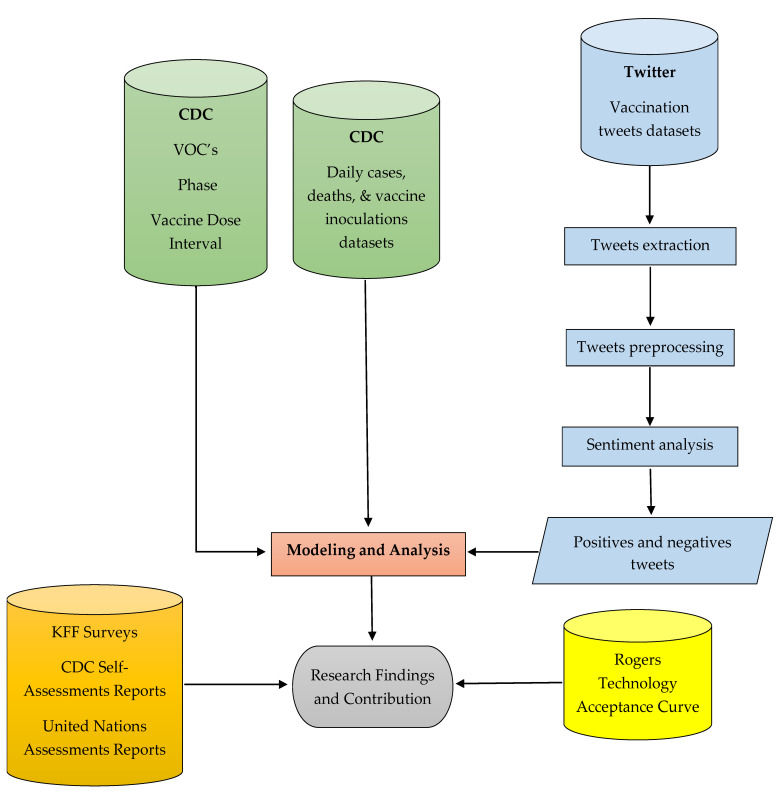
Research Methodology.

Figure 3 shows the relationships between the datasets used to conduct the research and analysis. Shown on the left side of the figure, the CDC issued virus and VOC alerts while the FDA approved three vaccine doses with the first dose and the second dose available for inoculation throughout the entire research window. The booster dose became available for general inoculations on 22 September 2021. Additionally, as shown in the center of the figure, the CDC reported daily a running total of vaccine inoculations, virus cases, and deaths. To complete the right side of Figure 3, Twitter datasets provide positive, negative, and neutral tweets about the virus and the vaccine. Twitter data extraction is discussed in more detail below. Finally, the bottom of the figure shows that the KFF vaccination survey outputs and Rogers Technology Acceptance Curve population segment descriptions provided third party analysis and benchmarks of public opinions and traditional behaviors that may relate to the research.

- CDC variant of concern announcement establish phases (Baseline, Delta, Omicron);
- CDC vaccine approvals establish dose intervals (first dose, second dose, booster dose);
- CDC case, deaths, and inoculation data collection and segmentation;
- Twitter data collection and sentiment analysis;
- Linear regression models;
- Correlation analysis.

### 3.1. Twitter Data Extraction

Twitter data may be extracted based on user location and demographics that exist on their profiles. Data extraction from Twitter focused on the United States users and their tweets about inoculation and vaccine hesitancy for the three COVID-19 vaccine types available (Pfizer, Johnson and Johnson, and Moderna). Users’ tweets were extracted from the Twitter feed using the technique described in Table 1 below, and were demonstrated by [25,26]. We built and coded our Tweepy library crawler following the [26] approach to extract tweets containing words identified in Table 2. “Tweepy is an open-source Python package that gives one a very convenient way to access the Twitter Application Programming Interface (API) with Python” [27] “in order to compose tweets, read profiles, and access your followers’ data and a high volume of tweets on particular subjects in specific locations” [28].

### 3.2. Modeling Construction

Modeling involved correlations and linear regressions to predict daily first dose, second dose, and booster vaccination inoculations. Regression independent terms included a constant associated with each dose type and variables associated with phase, daily virus cases, daily deaths, daily positive tweets, and daily negative tweets on day x. Respective regression equation outputs included first-dose vaccination inoculation for day x + 1, second-dose vaccination inoculation for day x + 1, or booster-dose vaccination inoculation for day x + 1.

## 4. Results

### 4.1. CDC Results and Analysis

As context to the research, on 31 May 2021, the day prior to the start of this research, the CDC reported that 173,531,874 (52.3% of a 331,800,000 population) had received first-dose inoculations. A total of 146,813,131 (44.2%) had received a second-dose inoculation, making them “fully vaccinated” at the time. Since the booster shot had not been authorized for the general public on 31 May 2021, only 9318 people had received the booster, representing 0% of the population.

**Figure 4:**
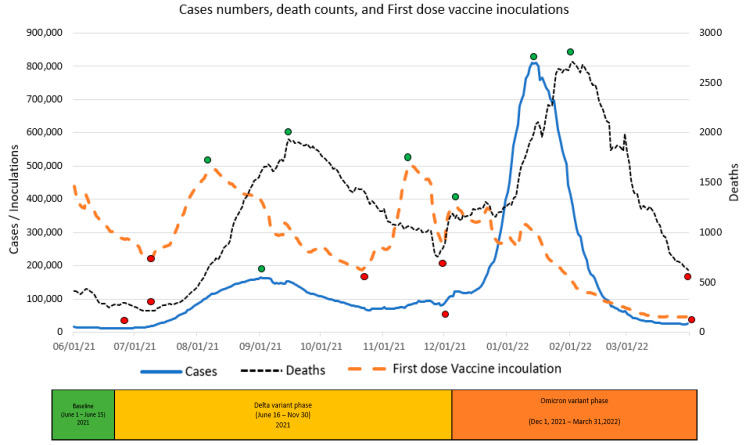
First-dose vaccine inoculations versus Virus Daily Cases & Deaths.

**Figure 5:**
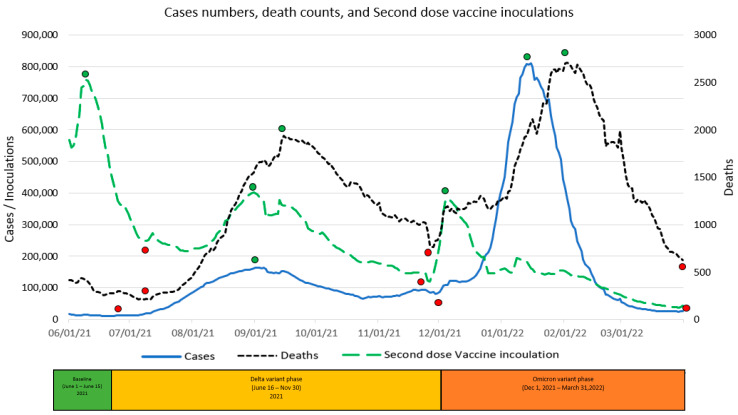
Second-dose vaccine inoculations versus Virus Daily Cases & Deaths.

**Figure 6:**
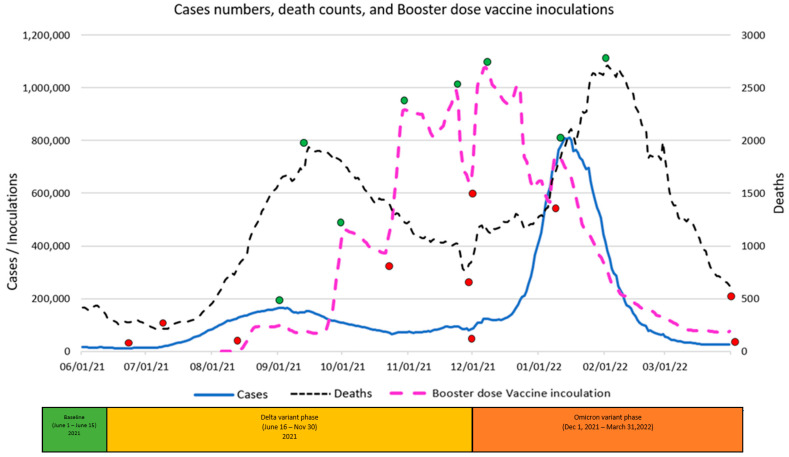
Booster-dose vaccine inoculations versus Virus Daily Cases & Deaths.

The graphic displays in Figure 4, Figure 5 and Figure 6 summarize daily virus cases and death counts and, respectively, the first, second, and booster doses reported by the CDC during the duration of the research. Beneath the graphic display of daily CDC data reported, the colored bar indicates the three phases of the pandemic identified by the CDC through their initial COVID-19 virus alert and subsequent Delta VOC and Omicron VOC alerts, as discussed in the Methods and Material section above.

Within each figure, a red dot indicates a trough inflection point and a green dot indicates a peak inflection point for a given curve. Common to all three figures are two virus case peaks that precede lagging death count peaks, corresponding, respectively, to the Delta and Omicron phases. Inoculation ups and downs for a given dose infers, respectively, a rising level of virus fear or rising level of vaccine hesitancy among the remaining populations for each dose type.

Common to all three inoculation curves is an overall decline toward zero for new inoculations at the end of the research timeframe. At the conclusion of the research, the CDC reported 256,144,043 (77.1%) first-dose, 219,319,838 (66.1%) second-dose, and 100,230,127 (45.7%) booster-dose inoculations. The declining inoculation percentages with each dose type as well as the failure of any of the dose types to exceed 78% coverage raises the notion of population segments with different levels of acceptance of new technology (e.g., different vaccine doses), as identified in the Technology Acceptance Curve (Rogers, 1995), where acceptance would be expressed as a sentiment. The variability in virus fear and vaccine hesitancy for each inoculation type (first, second, booster) varies significantly and is discussed in the next section in terms of correlations with objective virus case and death count measures. The nature and degree of change in inoculation behavior attributed to changing social sentiments are deferred to the Twitter Sentiment Results and Analysis section below.

### 4.2. Correlations between Inoculations and Virus Cases and Death Counts

A visual inspection of Figure 4 indicates a rapid drop in first-dose inoculations, virus cases, and virus deaths during the Baseline phase, at that time supporting the “eradication” theory. For the Baseline phase, correlation analysis confirmed large correlations between declining first-dose inoculations, declining death counts (0.886), and declining virus cases (0.766). In terms of the two theoretical premises, the correlations support the notion that the overall declining fear of sickness from the virus and declining fear of death from the virus resulted in declining inoculations among the remaining undosed population.

Despite the 15 June 2021 CDC Delta VOC alert and contrary to the theoretical expectation of an increase in the fear of the virus a VOC might cause, the public appeared to ignore the VOC as inoculations continued to drop even after the VOC. Inoculations also continued to drop inversely to rising virus cases (−0.872), until the inoculation trend reversed at an inflection point 23 days (8 July) into the Delta phase coincident with, not preceded by, an increase in the death count. The VOC alert, the long increasing virus cases, and the existence of a preceding increase in death count do not appear causal to the change in inoculation trend; the trend change is discussed below in the Twitter Sentiment Results and Analysis section. Between the inoculation trough and subsequent 8 July 2022 peak, large correlations were observed between rising first-dose inoculations and rising virus cases (0.987) as well as rising death counts (0.928). After the first peak and as also discussed in the Twitter Sentiment section below, inoculation behavior, inconsistently correlated with virus cases and death count, peaked before the Thanksgiving Holiday and again before the Christmas Holidays.

Based on a visual observation of the data, one might assume that the data exhibit an underlying persistent periodic signal similar to autocorrelation, which is typical of a harmonic frequency. As revealed in the regression equations below, up and down waves correlate with the Variant of Concern announcements, and not with an underlying persistent autocorrelation. Specifically, though the virus may mutate over time, mutations do not occur in a consistent pattern nor within a consistent timeframe. Further, biologically, the variants also tend to become less virulent over time to the point of becoming endemic, not pandemic. Less virulent variants cause less fear of the virus, resulting in fewer vaccinations. The tending toward zero may be noticed in the January through March data as each series persistently approach zero, which is not characteristic of autocorrelation. In summary, each series of data is dependent on factors to the degree correlated in the regression models below.

A visual inspection of Figure 5 reveals a second-dose peak of 758,476 on 9 June 2021 in the midst of the Baseline phase when virus cases and death count were both going down. This inverse correlation during the first part of this phase infers that a large segment of the population sought to be “fully vaccinated” despite the dropping virus cases and death counts. After the initial inoculation peak, second-dose inoculations fell until 29 July 2021, even though this was well past the 18 June 2021 virus trough and subsequent increase in virus cases. The inversely correlated precipitous drop in inoculations with rising virus cases potentially manifests higher levels of vaccine hesitancy among the remaining unvaccinated population segments. From 29 July 2021 to the 31 August 2021 inoculation peak, rising second-dose inoculations were highly correlated (0.943) with rising virus cases. After the 31 August 2021 peak, second-dose inoculations varied due to the sentiment factors discussed below.

A visual inspection of Figure 6 reveals that the vast majority of the booster-dose inoculations occurred entirely within the research timeframe and experienced multiple peaks with the highest daily peak of 1,078,908 booster-dose inoculations occuring on 7 December 2021 alone. Factors driving booster-dose inoculations are discussed in the Sentiment section below.

### 4.3. Twitter Sentiment Results and Analysis

Over the entire research timeframe, 949,529 tweets have been classified sentimentally. Sentiment analysis identified 326,124 tweets that indicated a positive viewpoint toward vaccines and 163,716 tweets that indicated a negative viewpoint toward vaccines, while 459,689 tweets indicted neutral sentiments toward vaccines.

**Figure 7:**
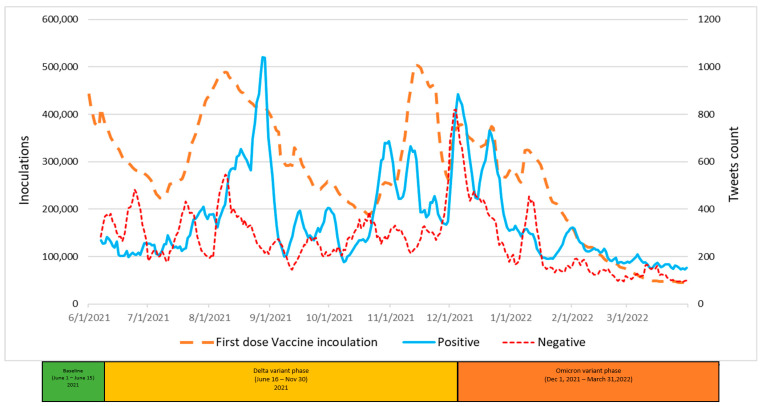
Positive and Negative Tweets toward First-Dose Vaccines and Vaccinations over Time.

A visual analysis of Figure 7 appears to show, and the analysis confirms, large positive correlations between Twitter positive tweets and first-dose inoculations across the entire Delta variant phase (0.607) and across the entire Omicron variant phase (0.804). Catalysts for tweet activity include CDC, FDA, and other Biden administration announcement events. Starting from July 2021, positive tweets increased slowly until the end of August 2021 when there was a significant jump in positive tweets. Positives tweet levels spiked again in September 2021 with approval events for the Pfizer booster dose for regular use, and again in October 2021, which was coincident with approval events associated with the Moderna booster and the approval of first-dose vaccinations for children. Negative tweets cited recent press reports on vaccine side effects, which likely increased the level of vaccine hesitancy sentiment. Nonetheless, paradoxically, a large correlation of 0.794 was observed between negative tweets and first-dose inoculations during the Omicron phase.

**Figure 8:**
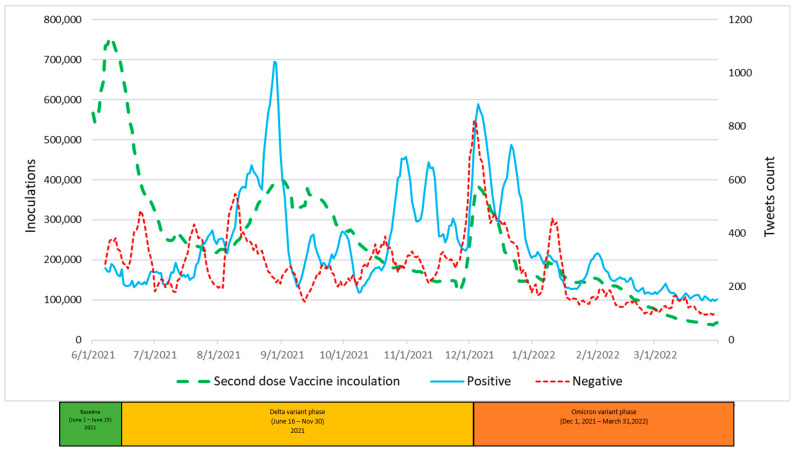
Positive and Negative Tweets toward Second-Dose Vaccines and Vaccinations over Time.

Visual analysis of Figure 8 also appears to show, and the analysis confirms, a large positive correlation (0.870) between positive tweets and second-dose inoculations during the Omicron phase. Paradoxically, a large correlation of 0.892 was observed between negative tweets and second-dose inoculations during the Omicron phase.

**Figure 9:**
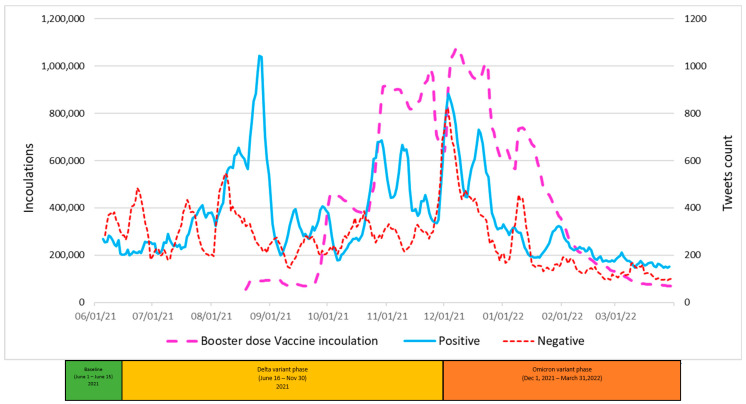
Positive and Negative Tweets toward Booster-Dose Vaccines and Vaccinations over Time.

Visual analysis of Figure 9 also appears to show, and the analysis confirms, a large positive correlation between positive tweets and booster-dose inoculations during a portion of the Delta phase (0.768) and across the entire Omicron phase (0.868). Paradoxically, a large correlation of 0.845 was observed between negative tweets and booster inoculations during the Omicron phase.

### 4.4. Regression Models Outcomes

**Figure 10:**
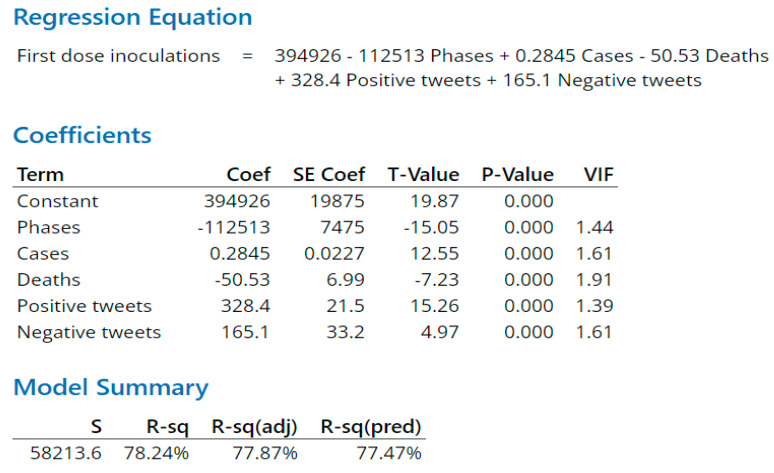
First-dose prediction model. *p* values are significant for all five x variables.

**Figure 11:**
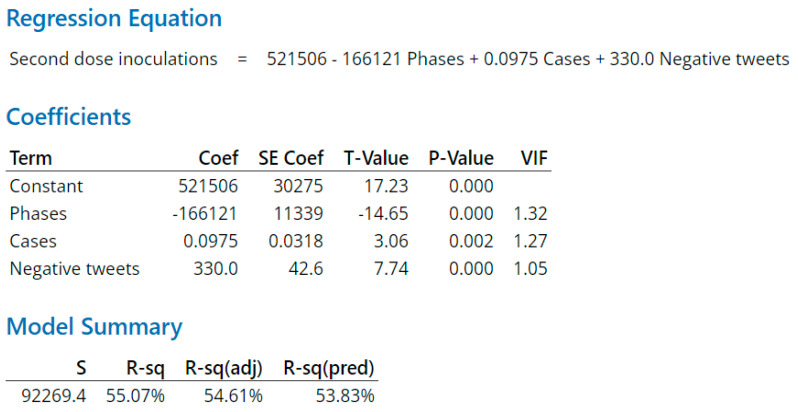
Second-dose prediction model. *p* values are significant for all three variables except the positive tweets and deaths variables which have been removed as their *p* values were not significant.

**Figure 12:**
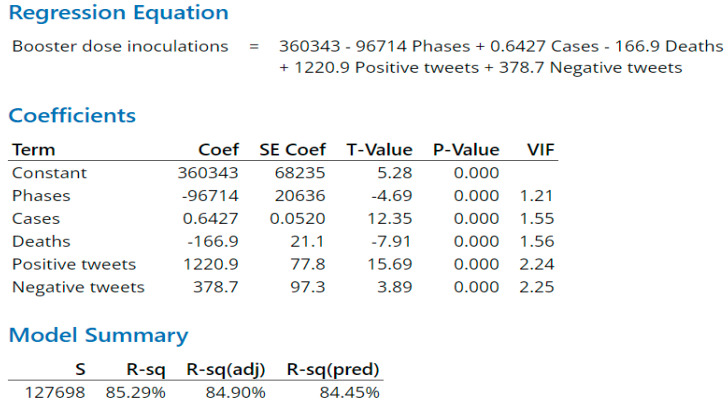
Booster-dose prediction model. *p* values are significant for all five variables.

As indicated in the Methods section, regression models for predicting daily first-dose, second-dose, and booster-dose vaccination inoculations quantify independent terms and their coefficients. The terms include a constant associated with each dose type and variables associated with phase, daily virus cases, daily deaths, daily positive tweets, and daily negative tweets on day x. The respective regression equation output calculates the first-dose vaccination inoculation for day x + 1 (Figure 10), second-dose vaccination inoculation for day x + 1 (Figure 11), or booster-dose vaccination inoculation for day x + 1 (Figure 12). R-Squared is a statistical measure that determines the proportion of variance in the dependent factor that can be explained by a regression model’s independent factors. When considering the importance of the factors, R-squared tends to reward overfitting a model with too many factors. Overfitted models lack predictive capability. Adjusted R-squared and predicted R-squared address too many factors and overfitting by, respectively, adjusting for the number of terms in the model and evaluating how well the model predicts by repetitively removing data points and recalculating the regression.

First- (Figure 10) and booster-dose (Figure 12) inoculation models both had large predictive R-squares of 77.47% and 84.45%, respectively. First-dose and booster-dose inoculation models had in common: all factors of interest, each factor was statistically significant in predicting daily inoculations in both models, and each corresponding factor was directionally the same in each model. Death counts for both inoculation models actually had a negative impact on inoculation counts, inferring that death counts were a lagging indicator. Differences between the first and booster inoculation models emerge with sentiment-variable coefficients. Combined sentiment variables (positive and negative tweets) impact the booster-dose inoculation model 3.23 times as great as the same variable impacts the first-dose model. Of note, during the research timeframe, CDC and Biden administration announcement events occurred more often in association with the first and booster doses, with only one event associated with the second dose (Figure 11). Tweets increase around such events, with positive tweet spikes observed in association with first- and booster-dose events. Negative tweets increased with smaller spikes than positive tweets during events except during the Baseline phase, where the negative tweets were at the same level of positive tweets.

The second-dose inoculation regression model (Figure 11) had a moderate predictive R-square of 53.83%. Of immediate note between the three regression equations is the 32% and 44.7% respective differences between the 394,926 first- and 360,343 booster-dose equation constants and the 521,506 second-dose constant. The significantly higher second-dose constant highlights the aforementioned importance of being “fully vaccinated” for significant segments of the population. Being “fully vaccinated” drives behavior for this segment of the population even to the point of getting the second dose in the Baseline phase despite numerous, although later proven false, indications of a waning pandemic. Similarly, the much larger negative coefficient of the “phase” variable in the second-dose equation infers a rapid drop off in inoculations across phases, highlighting this population segment’s urgency during the Baseline phase in completing the series. Interestingly, neither death count nor positive tweets had a significant impact on second-dose inoculations. Cases made a modest contribution toward inoculations. Negative tweets paradoxically made a significant contribution to inoculation, inferring resoluteness of or perhaps even indifference or defiance toward negative social media by this population segment for getting the second dose and becoming “fully vaccinated”.

For the booster-dose model, the positive tweets were three times as many as negative tweets. Drivers of the differences were: the Pfizer booster approval in September 2021 and Moderna & J.J booster-dose approvals in October 2021. In addition, the booster-dose interval constant was lower in part due to the booster never being authorized for children, but this also indicates a lower inclination on the part of subjects to get the booster than observed with either the first dose or second dose.

## 5. Discussion

### 5.1. Kaiser Family Foundation Surveys and CDC Self-Assessment Report

The results from the Kaiser Family Foundation (KFF) surveys indicate major reasons for getting the COVID-19 vaccine during our research timeframe, of which were: an increase in cases due to the Delta variant (39%), concern about hospitals filling up (38%), and knowing someone that became seriously ill or died from COVID-19 (36%). These findings appear to generally support the regression factors common to the first-dose and booster-dose regression models and contribute to their large predictive R-square (“Surging delta variant cases, hospitalizations, and deaths are biggest drivers of recent Uptick in US COVID-19 vaccination rates”, 2021) [37]. For this research, inconsistencies are most notable between KFF and the moderately predictive, second-dose regression model. Specifically, and most notably, there is an absence of the death factor from the list of second-dose regression factors. The moderate, rather than large, predictive R-square of the second-dose regression model is likely an artificiality of the limitations of the research timeframe. Specifically, for the large predictive R-squares for the first-dose and booster-dose regression models, the vast majority or the entire total of inoculations taken within our timeframe occurred outside the Baseline phase. For the second-dose regression model, a much larger percentage of second-dose inoculations taken during our timeframe occurred during the Baseline phase. Thus, motivation before the Baseline phase is more likely to have driven second-dose inoculation behavior than driven either first-dose or booster-dose inoculations.

The CDC has carried out numerous self-assessment reports of their role in the COVID-19 pandemic. Ref. [38] studied the second-dose phenomenon and in part found that behavior differed by population segments driven largely by age. Specifically, “Compared with first-dose recipients 18–39 years of age, recipients 40–64 and >65 years of age were less likely to have missed a second dose. Persons in older age groups had more time to complete their primary series, given the prioritization when COVID-19 vaccine first became available. Older adults also are at higher risk for severe COVID-19 illness and may have been more motivated to become fully vaccinated (14,15)”. If the findings from Meng et al. are accurate, the findings support the notion that the most likely factor adversely impacting the second-dose regression model resulting in a moderate rather than large predictive R-square was that the older segment of the population disproportionately got inoculations during the Baseline phase. As Meng et al. also further indicate, older population segments may have been driven to become “fully vaccinated” with the second dose by fear of “severe illness” or death. Since the pandemic began in the United States in March 2020 and without knowledge of the Delta and Omicron VOCs, this fear would have been accumulated well before the Baseline phase and before our research timeframe. Future research that included data reaching back to March 2020 would likely significantly change second-dose regression model factors and weights to be more in line with first-dose and booster-dose regression models. Additionally, future research may improve the predictive R-square of the regression equations by including a variable for population segments, possibly identified by age and other demographics that may impact vaccine acceptance. Furthermore, the future research can improve the prediction accuracy by using machine learning modeling approaches, which have been proven to be more powerful and effective than conventional approaches [39,40].

A KFF survey also solicited feedback on sentiments about vaccine side-effect concerns and the resulting vaccine hesitancy (Personal Concerns About COVID-19 Vaccination) [41]. For the booster dose, KFF identified significantly different sentiments among vaccinated and unvaccinated population segments. Specifically, 78% of the vaccinated respondents indicated that the booster dose “shows that scientist are continuing to find ways to make vaccines more effective” [42]. In contrast, 71% of the unvaccinated respondents indicated that the booster dose “shows that the vaccines are not working as well as promised”. KFF also indicated that the Omicron variant only motivated about 12% of the unvaccinated to get their first dose, while 87% remained unconvinced [43]. In contrast, among vaccinated adults who had not gotten a booster, 54% indicated that the Omicron variant made it “more likely” to “get a booster shot”, while 46% disagreed.

### 5.2. Considering Vaccine Acceptance Rates in Light of Rogers Technology Acceptance Curve

Given the aforementioned segmentation of the population, this research would be remiss not to acknowledge similarities between population acceptance of and hesitancy toward vaccines and the Rogers Technology Acceptance Curve. Specifically, the Rogers Technology Acceptance Curve identifies five segments the US population and characterizes 2.5% as Innovators in accepting technology, 13.5% as the Early Adopters, 34% as the Early Majority, 34% as the Late Majority, and 16% as Laggards. Laggard traits include skeptical, resistance to change, and wary of accepting new technology. Assuming that each inoculation is a new technology experiencing the Rogers estimates of acceptance, laggards would represent the segment of the population predisposed toward vaccine hesitancy. This assumption infers that approximately 84% of the population might voluntarily accept a first-dose inoculation within the timeframe of the pandemic. As of 7 February 2023, and remarkably consistent with the Rogers estimate of 84% acceptance, the CDC reports that 85.5% of the US population 5 years of age or greater are inoculated with one dose [38]. If a second-dose inoculation is viewed as another voluntary acceptance challenge, then the Rogers estimation of 84% of the first-dose recipients would yield that an estimate of 70.6% of the total population will receive a second-dose inoculation. As of 7 February 2023 and remarkably consistent with the Rogers estimate of 70.6% acceptance, the CDC reports that 73.2% of the US population 5 years of age or greater are inoculated with one dose [38]. The respective 1.5% and 2.6% higher observed inoculation rates over that estimated by the Rogers Curve may be due to vaccination mandates imposed by government and/or employers. Unfortunately, the Rogers statistical booster-dose estimation is confounded by a replacement of the original booster with the updated (bivalent) booster dose. Further, as of 7 February 2023, the CDC only reports updated (Bivalent) booster data, not the original booster data discussed herein. The 31 March 2022 CDC reported 100,230,127 (45.7%) original booster inoculations; applying Rogers 84% acceptance rate to the 70.5% who actually accepted the second-dose inoculations yields an expected 59.3% acceptance rate for booster inoculations among the total population. Clearly, the observed 45.7% acceptance rate is well below the 59.3% estimated rate. Future research may reveal the reasons for the inoculation shortfall, but the shortfall is likely due to the timeframe limitations of the experiment or timeframe limitations due to the replacement of the booster with the updated booster, but may also be due to rising vaccine hesitancy or fewer mandates.

### 5.3. Social Media Manipulation, Sentiment Analysis, Twitter Files, and United Nations Assessment Reports

While we believe our sentiment analysis approach is sound and the results are accurate to the degree cited above, manipulation by government agencies or large corporations may have an impact on social media content and may therefore limit sentiment analysis where such manipulation occurs. Social media content impacts sentiment. Revelation of content manipulation will likely undermine the confidence in content found on social media and thereby will undermine the value of social media sentiment analysis. As an example, with the acquisition of Twitter by Elon Musk, a number of independent journalists investigated governmental and industrial manipulation of social media and released reports termed the “Twitter Files”. Among those investigations, David Zweig released the 40-tweet Twitter Files report titled, “How Twitter Rigged the COVID Debate” [44]. Zweig identified in that report “that both the Biden and Trump administrations pressured Twitter and other social-media platforms to elevate content that fit their narratives and to suppress information that didn’t”. One of the more important and concerning findings was actual interference in free speech by the silencing of Alex Berenson, a critic of the Biden administration COVID policies. Specifically, “Berenson’s Twitter account was suspended hours after Biden alleged that social-media companies were “killing people” for allowing vaccine misinformation. Berenson later sued and eventually settled with Twitter”. Further, Lee Fang revealed how “the pharmaceutical industry lobbied social media to shape content” related to the COVID vaccine [45]. Even Pfizer board member Dr. Scott Gottlieb flagged tweets questioning COVID vaccines [46]. An August 2021 email Gottlieb sent to Twitter’s senior public policy manager Todd O’Boyle flagging a tweet written by former Trump administration official Dr. Brett Giroir is but one example. Giroir had written “It’s now clear #COVID19 natural immunity is superior to #vaccine immunity, by ALOT. There’s no scientific justification for #vax proof if a person had prior infection”. “This is the kind of stuff that’s corrosive”, Gottlieb told O’Boyle. “Here he draws a sweeping conclusion off a single retrospective study in Israel that hasn’t been peer reviewed. But this tweet will end up going viral and driving news coverage”. According to Berenson, O’Boyle forwarded Gottlieb’s email to Twitter’s “Strategist Response” team, writing “Please see this report from the former FDA commissioner”. Giroir’s tweet was later slapped with a “misleading” label and blocked any ability to like or share the tweet, telling Twitter users “Learn why health officials recommend a vaccine for most people”.

Besides the directed manipulation of social media content by governments and industry, this research revealed that CDC and Biden administration announcements and events precipitated considerable Twitter tweets. The nature of the tweet response directly impacted the regression equations. Thus, two recent United Nation reports stating, “scientist in China, the US and the UK have been accused of deliberately covering up the origins of the coronavirus outbreak” [47], may also undermine social media sentiment analysis in the future by creating further distrust. Epidemiologists Colin Butler, from the National Centre for Epidemiology and Population Health in Canberra, Australia, and Delia Randolph, from the University of Greenwich in London, were responsible for the reports. Both concluded that high-risk experiments being carried out in the Chinese city of Wuhan were shrouded in a cloak of suspicious secrecy, deception, and conflicts of interest. They argued that this was ‘implemented not only by China but also by Western funding agencies and influential Western scientists’.

While the sentiment analysis in this research did not consider the undermining of the confidence of the fair, accurate, and equitable publishing of social media content, future research must consider how potential social media content manipulation impacts behavior.

## Figures and Tables

**Table 1:** Twitter Data Extraction, Preprocessing, and Sentiment analysis Steps.

Step	Description
Vaccination tweets extraction	Using Tweepy library to extract tweets from the Twitter API that relevant to COVID-19 vaccine and vaccinations keywords [9,26]. In addition, using the geolocation features to extract tweets that were posted by the USA users.
Tweets preprocessing	The retweets and URLs were removed in the preprocessing step, emojis were converted into words, and the dataset was cleaned. We also removed stop words and performed tokenization. Stemming and lemmatization were carried out as well [29,30,31].
Sentiment analysis	Using Vader library to classify tweets into positive, neutral, and negative [32,33,34,35,36], where tweets classifications are defined as follows: - Positive tweets represent the opinions that in favor of taking the COVID-19 vaccines - Negative tweets represent the opinions that are against or hesitant to take the vaccines. - Neutral tweets represent the opinions that are not in favor or against the vaccines, where tweets cannot represent positive neither negative opinions.

**Table 2:** Vaccination keywords used to extract the Twitter datasets.

Vaccine	Keywords	Timeframe
Pfizer-BioNTech vaccine	Pfizer, Pfizer-BioNTech, BioNTechpfizer, vaccine, vaccination, dose	1 June 2021–31 March 2022
Johnson & Johnson’s COVID-19 Vaccine	Johnson & Johnson, Johnson and Johnson, Janssen, Janssen, vaccine, vaccination, dose	1 June 2021–31 March 2022
Moderna vaccine	Moderna, Moderna_tx, Moderna-NIAID, NIAID, NIAID-Moderna, vaccine, vaccination, dose	1 June 2021–31 March 2022

## Data Availability

Not applicable.
